# Re-appointment

**Published:** 1874-01

**Authors:** 


Reappointment.—Dr. E. C. W. O'Brien has been reappointed Health Physician by the new Board of Health. The appointment is one which will meet with approval, as the Doctor has shown during his terms of office that he is fully awake to the duties of his position. A better selection could not well be made.

———o———

We would call attention to the circular addressed to the Medical Profession at large in our Miscellaneous Department. The object is one in which all will sympathize, and we hope to see a lasting and appropriate monument erected to the memory of those faithful martyrs to the cause of humanity.

